# Changes in specific serum biomarkers during the induction of prostatic hyperplasia in dogs

**DOI:** 10.1186/s12917-019-2201-5

**Published:** 2019-12-05

**Authors:** Kamran Golchin-Rad, Asghar Mogheiseh, Saeed Nazifi, Mohammad Saeed Ahrari Khafi, Nooshin Derakhshandeh, Mohammad Abbaszadeh-Hasiri

**Affiliations:** Department of Clinical Sciences, School of Veterinary Medicine, Shiraz University, P.O.BOX 7144169155, Shiraz, Fars Iran

**Keywords:** Prostate specific antigen, Dog, Canine prostate specific esterase, Prostatic hyperplasia, Testosterone, Estrogen

## Abstract

**Background:**

Prostatic hyperplasia (PH) is one of the most important disorders in intact dogs. In this study, we aimed to induce PH experimentally using the combination of testosterone and estrogen and evaluate important factors associated with this disease.

**Results:**

The results showed that in the induction group, prostate volume and prostate specific antigen (PSA) concentration increased significantly on day 21 onwards compared to those of the control group. Canine prostatic specific esterase (CPSE) and dihydrotestosterone (DHT) concentrations increased significantly on day 42 onwards while the testosterone levels increased on day 63. In addition, prostatic acid phosphatase (PAP) concentration did not change significantly in the control and induction groups. Biochemistry profiles and hematologic factors were measured for monitoring the function of liver and kidney, and there were no adverse effects following the induction of PH.

**Conclusions:**

It seems that testosterone and estrogen administration led to prostatic hyperplasia during 2 months. Investigating the size of the prostate, accompanied by prostate markers including CPSE, PSA, DHT, and testosterone, is helpful for the PH diagnosis. However, further studies should be carried out on PAP.

## Background

Prostatic hyperplasia (PH) can only develop spontaneously in dogs and humans. In intact male dogs, it is mostly a natural consequence of aging and increasing concentrations of dihydrotestosterone, which leads to an increase in cell number (hyperplasia) and cell size (hypertrophy) [1–8]. During the aging process (from 5 to 9 years old), almost 80–95% of dogs are affected by PH [1–5, 9].

However, clinical signs associated with PH will not develop until the enlarging prostate presses rectum and urethra and causes tenesmus or hyperplastic tissue with increased vascularity leads to hematuria. Consequently, serous to sanguineous urethral discharge and hemospermia may be observed [3, 4, 9].

Prostatic gland development and its proper function require testosterone production in the testicular tissue. The factors that can initiate PH and its development are testosterone (T), dihydrotestosterone (DHT), growth factors and intraprostatic estrogen. The overproduction of DHT, a hormone which collaborates with receptors in the prostate, is a pivotal factor in PH pathogenesis and is derived from testosterone by the prostatic enzyme, type II of 5-alpha reductase. Testosterone secretion, as well as the number of receptors and the ratios of prostatic cell growth compared to the prostatic cell death, increases as time goes by. In addition, PH is a predisposing factor to prostatic inflammation [10–12].

PH can be diagnosed by history, clinical signs, the prostatic contour through rectal palpation, prostatic size detected by radiography, prostatic volume and parenchyma detected by ultrasonography, semen culture and cytology, and biopsy by the gold standard test [13]. A large prostate with homogeneous echogenicity revealed in ultrasound examination. Also, small cysts may be observed in some prostate gland affected by PH [10].

There are some significant biomarkers of the reproductive tract of male dogs like canine prostate-specific esterase (CPSE), prostatic acid phosphatase (PAP), and prostate-specific antigen (PSA), which can be evaluated for the diagnosis of PH. Biomarkers are biologic substances in serum and seminal plasma which could be synthesized either by normal or abnormal tissues of the organism. These markers can be used for diagnosing pathological conditions [14]. CPSE is a serine protease which is secreted under the control of sex hormones by prostatic epithelial cells. CPSE comprises more than 90% of the protein in the prostatic fluid and is present in seminal plasma and blood. When hyperplasia occurs in the prostatic cells, the serum concentration of CPSE increases [15]. An excellent indicator of PH in dogs is the serum concentration of CPSE [16, 17]. Hormones can regulate CPSE and PSA, and their serum and seminal plasma levels and activities are reduced following the decrease of the serum testosterone activities. CPSE has been detected in normal (canine prostatic cells, seminal fluid) and abnormal conditions (neoplastic and hyperplastic prostatic tissue) [18, 19]. Usually, the recurrence of prostatic carcinoma is evaluated according to serum PAP and PSA levels in men; however, in dogs, information about these biomarkers is still inconclusive [20]. Like men, the concentration of PAP in dogs is dependent on the hormone, but, its dependency on age is variable.

Quantitative alterations of PAP in dogs are less significant than in humans; however, these alterations play a key role in the assessment of the secretory activity of prostatic epithelial cells [21]. Because the PAP concentrations are high in the inflammation of the prostate, prostatic massage, PH, and digestive tract cancer, PAP should not be considered as a specific marker for prostatic diseases [22]. PSA is a smaller molecule than PAP and is diffused more easily via the basal membrane of prostatic acinar cells. Thus, PSA concentration is more sensitive than that of PAP while the specificity of the PAP level is greater than that of PSA [23, 24]. Monitoring PAP values in normal, PH, and prostatic adenocarcinoma dogs revealed that it was not possible to differentiate prostatic neoplasia from other conditions based on the low levels of PAP. However, higher levels of PAP can be indicative of cancer cases [22].

The purpose of this study was to monitor and evaluate the changes in the size of the prostate manifested in the ultrasonographic examination and serum biomarkers during the experimental induction of PH in dogs.

## Results

### Ultrasonography examination

**Fig. 1:**
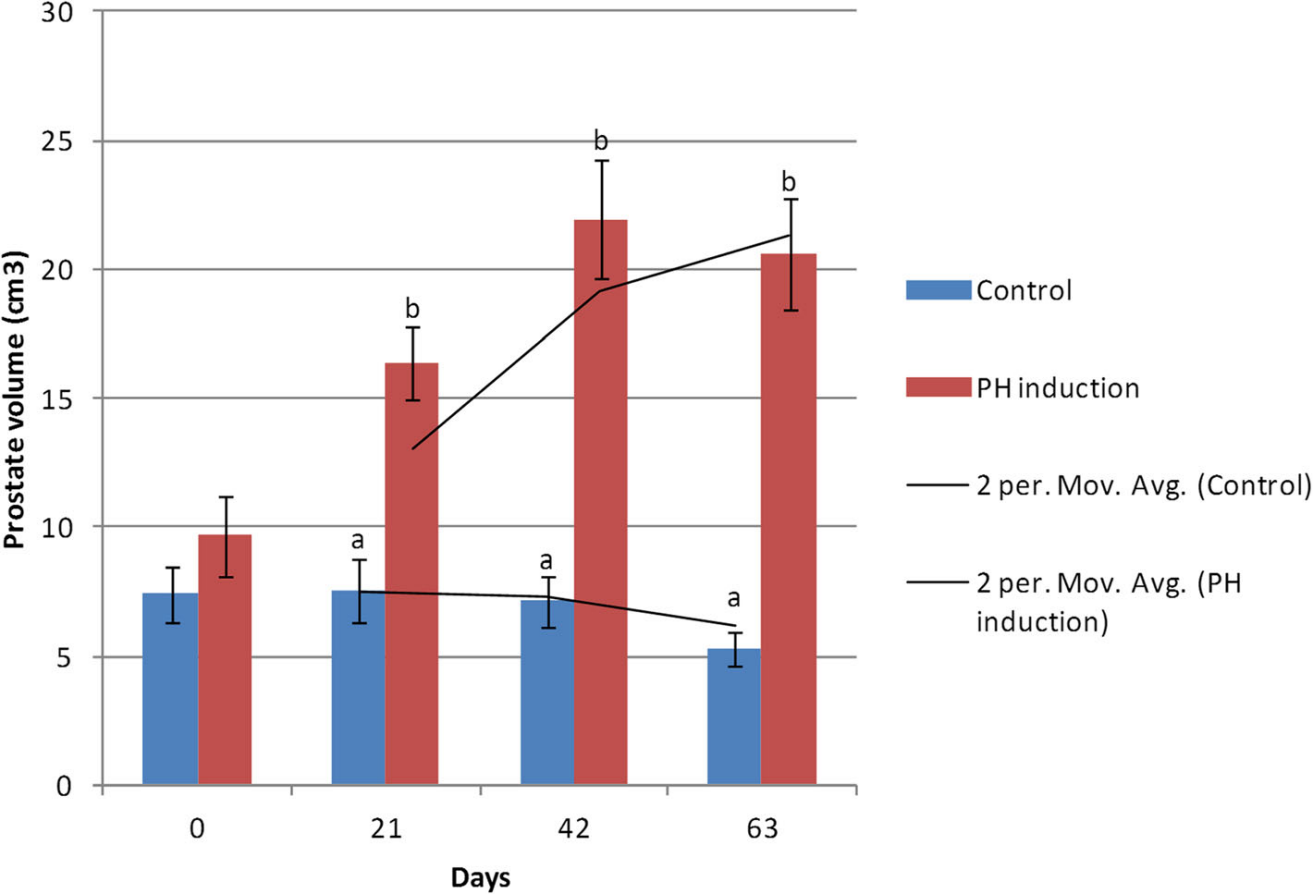
Changes and comparison of the prostatic volume during the induction of PH with testosterone and estrogen in the control and PH-induced groups of intact dogs (*n* = 20). Significant differences between the groups are indicated with different letters above the columns

In the control group, the minimum volume of the prostate was 5.28 ± 0.66 cm^3^ on day 63, and the maximum volume was 7.52 ± 1.22 cm^3^ on day 21. In the induction group, the minimum volume of the prostate was 9.66 ± 1.52 cm^3^ on day 0 while the maximum diameter was on day 42 (21.92 ± 2.31 cm^3^). The mean volume of the prostate in the control group was 6.83 ± 1.00 cm^3^ whereas it was 17.12 ± 1.85 cm^3^ in the induction group. The prostate volume increased from 9.66 to 20.59 cm^3^ (113.18%) from day 0 to day 63 of the study in the induction group (Table 1). The average volume of the prostate changed during the induction, and it was significantly different in terms of time and group factor (*P* < 0.0001). The comparison between the groups at any time suggested that from day 21 (*P* = 0.0004) onwards, the size of the prostate was different between the groups (*P* < 0.0001 on day 42 and 63). Over time, the prostate volume of the control group did not change, but significant changes were observed in the induction group in terms of the prostatic volume between day 0 vs. 21 (*P* = 0.001), 42 (*P* < 0.0001), 63 (*P* < 0.0001), and day 21 vs. 42 (*P* = 0.007; Fig. 1).

**Table 1:** Changes in the serum prostatic biomarkers during the study from day 0 to day 63 in the control and PH-induced dogs

Prostatic biomarker	Group	Day 0	Day 63	Changes (%)
Prostate volume (cm^3^)	Control (*n* = 10)	7.40	5.28	−28.64
	PH induction (*n* = 10)	9.66	20.59	113.18
CPSE (ng/ml)	Control (*n* = 10)	71.16	70.55	−0.85
	PH induction (*n* = 10)	78.19	135.96	73.86
PSA (ng/dl)	Control (*n* = 10)	0.0052	0.0053	1.92
	PH induction	0.0066	0.0508	669.69
PAP (U/L)	Control (*n* = 10)	2.666	2.665	−0.037
	PH induction (*n* = 10)	2.656	2.668	0.45
Testosterone (pg/ml)	Control (*n* = 10)	0.484	0.488	0.82
	PH induction (*n* = 10)	0.654	0.795	21.55
DHT (pg/ml)	Control (*n* = 10)	156.88	153.47	−2.17
	PH induction (*n* = 10)	169.67	179.85	5.99

### CPSE

**Fig. 2:**
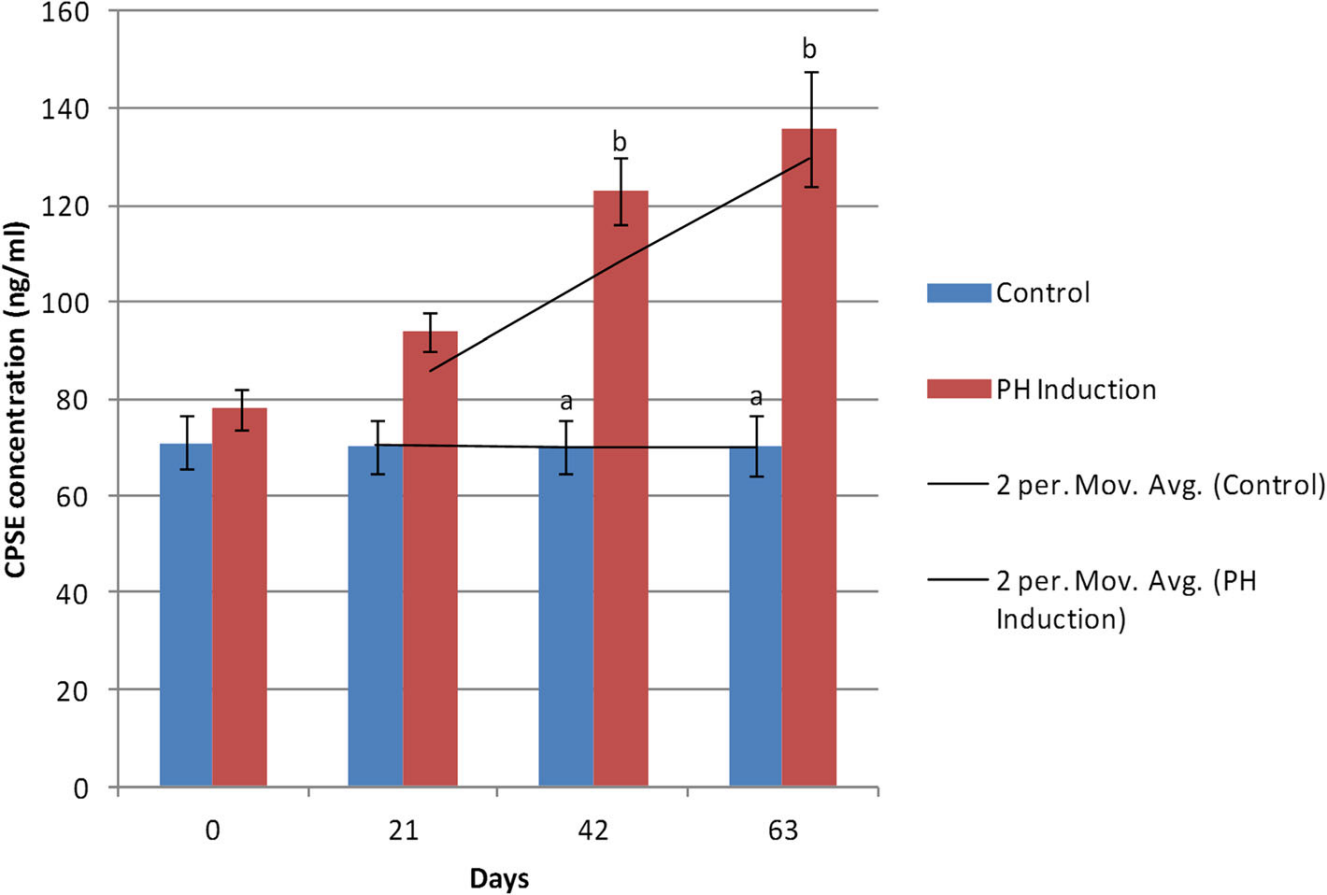
Changes and comparison of blood serum CPSE concentration during the induction of PH with testosterone and estrogen in the control and PH-induced groups of intact dogs (*n* = 20). Significant differences between the groups are indicated with different letters above the columns

In the control group, a minimum concentration of CPSE (70.20 ± 5.86 ng/ml) was seen on day 21, and the maximum level (71.16 ± 5.75 ng/ml) was observed on day 0. The mean CPSE concentration in the control group was 70.63 ± 5.99 ng/ml while it was 107.73 ± 6.70 ng/ml in the induction group. In the induction group, the minimum level of CPSE (78.19 ± 4.11 ng/ml) was on day 0 and the maximum level was on day 63 (135.96 ± 11.76 ng/ml). The CPSE concentration increased to 73.86% (from 78.19 to 135.96 ng/ml) from day 0 to day 63 of the study in the induction group (Table 1). In the comparison made between the control and the induction groups, no significant difference was observed on days 0 and 21 while significant differences were found on days 42 and 63 (*P* < 0.0001). When different days were compared with regard to the control group, the results showed no significant difference between them. Also, in the induction group, there were no significant changes in the CPSE concentrations between day 0 vs. 21 and day 42 vs. 63 while there were significant changes on days 0 vs. 42 (*P* = 0.0004), 0 vs. 63 (*P* < 0.0001), 21 vs. 42 (*P* = 0.03), and 21 vs. 63(*P* = 0.0009; Fig. 2).

### PSA

**Fig. 3:**
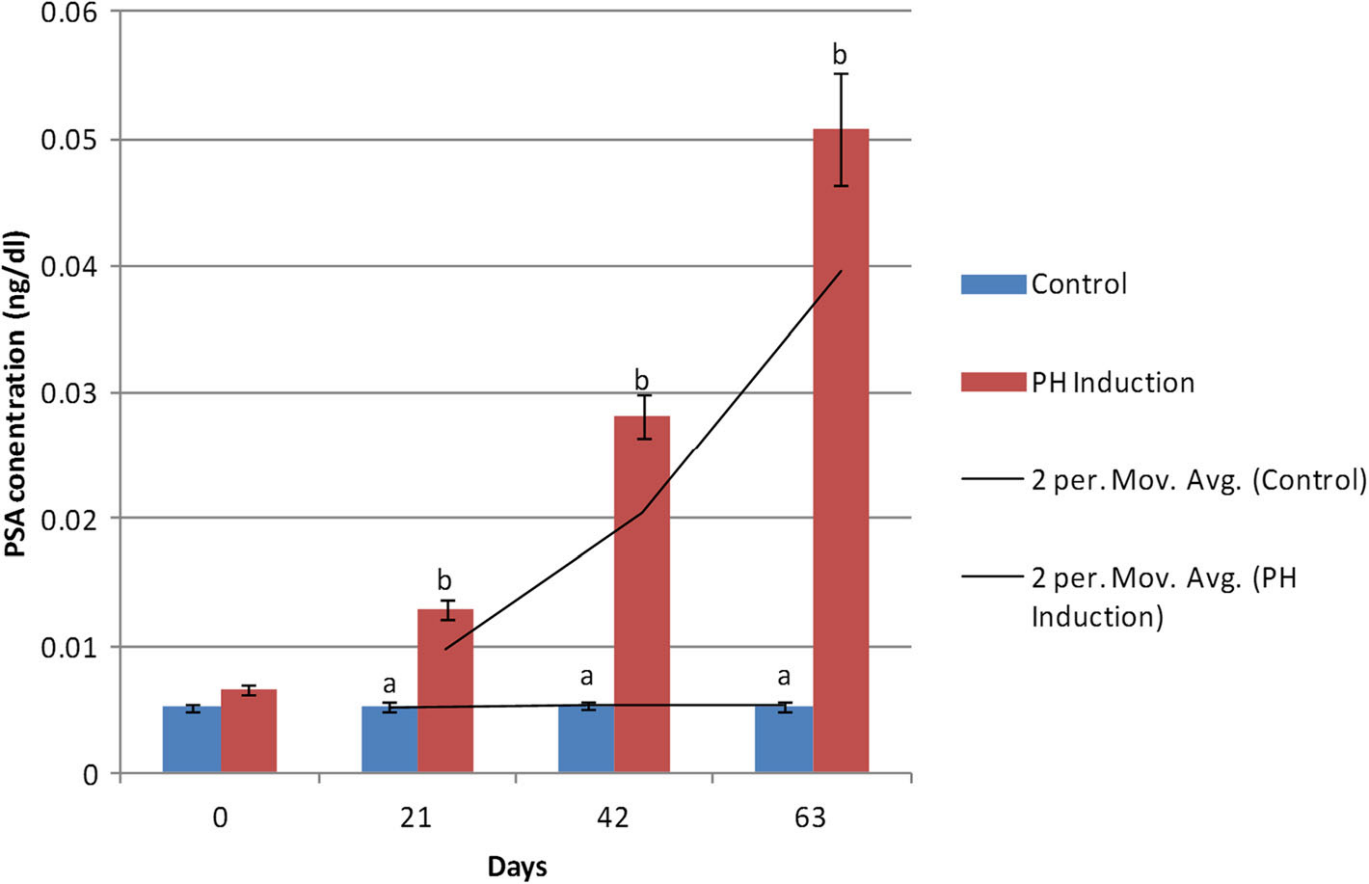
Changes and comparison of serum PSA concentration during the induction of PH with testosterone and estrogen in the control and PH-induced groups of intact dogs (*n* = 20). Significant differences between the groups are indicated with different letters above the columns

The lowest level of PSA (0.0052 ± 0.0002 ng/dl) in the control group was seen on day 0 while the maximum PSA concentration (0.0053 ± 0.0003 ng/dl) was observed on day 63. In the induction group, the minimum PSA level (0.0066 ± 0.0004 ng/dl) was on day 0 and the maximum concentration (0.0508 ± 0.0043 ng/dl) was on day 63. The mean concentration of PSA in the control and induction groups was 0.0052 ± 0.0003 ng/dl and 0.0246 ± 0.0018 ng/dl, respectively. The mean level of PSA increased from 0.0066 to 0.0508 ng/dl (669.69%) from day 0 to day 63 of the study in the induction group (Table 1). There was no significant difference between the control and induction groups on day 0 while there were significant changes in the PSA level on days 21 (*P* = 0.0077), 42 (*P* < 0.0001), and 63 (*P* < 0.0001). There were no significant differences between the days of sampling in the control group, but there were significant changes in the induction group on day 0 vs. 42, 0 vs. 63, 21 vs. 42, 21 vs. 63, and 42 vs. 63 (*P* < 0.0001; Fig. 3).

### Pap

**Fig. 4:**
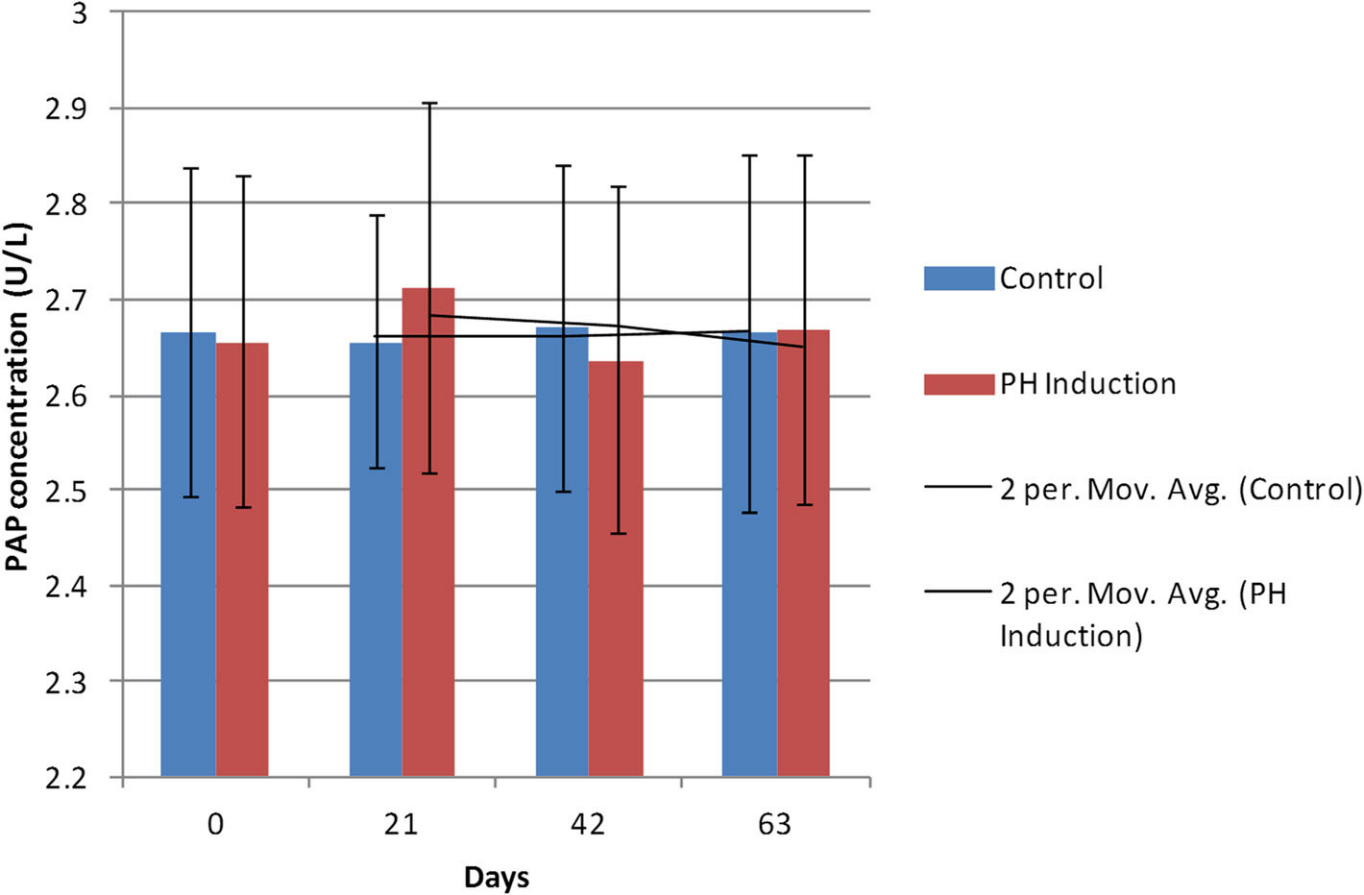
Changes and comparison of serum PAP concentration during the induction of PH with testosterone and estrogen in the control and PH-induced groups of intact dogs (*n* = 20). Significant differences between the groups are indicated with different letters above the columns

There were not any significant differences in any of the comparisons made between the PAP levels during the induction period. In the control group, the minimum concentration of PAP (2.65 ± 0.13 U/L) was seen on day 21, and the maximum PAP level (2.67 ± 0.17 U/L) was observed on day 42. In the induction group, the minimum PAP level (2.63 ± 0.18 U/L) was seen on day 42 and the maximum concentration (2.71 ± 0.19 U/L) was on day 21. The mean PAP concentration was 2.66 ± 0.16 U/L in the control group and 2.66 ± 0.18 U/L in the induction group. The percent of changes observed in the PAP concentration was 0.45% from day 0 to day 63 in the induction group (Table 1). The comparison between the groups showed no significant differences (*P* > 0.99). In the control and induction groups, there were no significant changes between the days of sampling (*P* > 0.99; Fig. 4).

### Testosterone

**Fig. 5:**
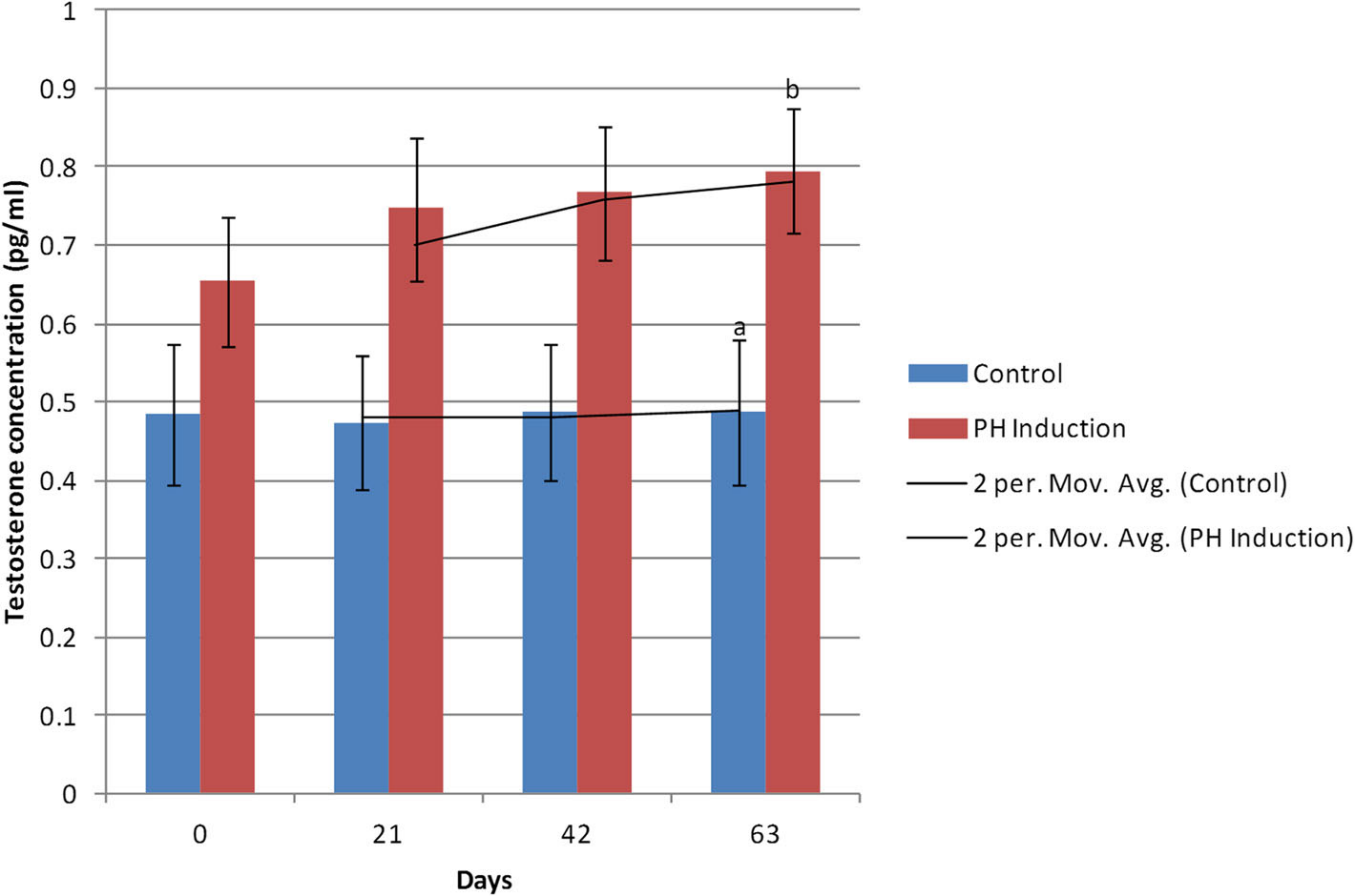
Changes and comparison of serum testosterone concentration during the induction of PH with testosterone and estrogen in the control and PH-induced groups of intact dogs (*n* = 20). Significant differences between the groups are indicated with different letters above the columns

In the control group, the maximum (0.48 ± 0.09 pg/ml) and minimum (0.47 ± 0.08 pg/ml) concentrations of testosterone were seen on day 42 and 21, respectively, whereas in the induction group, the minimum testosterone level (0.65 ± 0.08 pg/ml) was on day 0 and the maximum concentration of testosterone (0.79 ± 0.07 pg/ml) was on day 63. The mean testosterone concentration was 0.48 ± 0.08 pg/ml in the control group and 0.74 ± 0.08 pg/ml in the induction group. The testosterone level increased to 21.55% (from 0.65 to 0.79 pg/ml) from day 0 to day 63 of the study in the induction group (Table 1). There was a significant difference between the control and induction groups only on day 63 (*P* = 0.03; Fig. 5).

### DHT

**Fig. 6:**
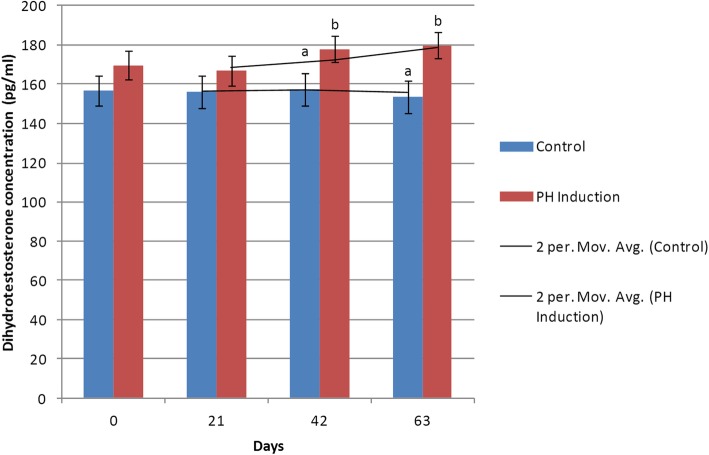
Changes and comparison of dihydrotestosterone concentration during the induction of PH with testosterone and estrogen in the control and PH-induced groups of intact dogs (*n* = 20). Significant differences between the groups are indicated with different letters above the columns

In the control group, the maximum DHT concentration (157.57 ± 8.16 pg/ml) was seen on day 42 while the minimum level (153.47 ± 8.17 pg/ml) was observed on day 63. In the induction group, the maximum concentration of DHT (179.85 ± 6.83 pg/ml) was seen on day 63 and the minimum level of DHT (166.87 ± 8.07 pg/ml) was observed on day 21. The mean concentration of DHT was 156.04 ± 24.60 pg/ml in the control group and 173.60 ± 7.07 pg/ml in the induction group. The level of DHT increased from 169.67 to 179.85 pg/ml (5.99%) from day 0 to day 63 of the study in the induction group (Table 1). The overall comparison between the control group and the induction group indicated that their differences were not significant on days 0 and 21, but on days 42 (*P* = 0.04) and 63 (*P* = 0.006), their differences were significant. Also, there were no significant differences between different days of sampling in either the control or induction group (Fig. 6).

### Correlation and regression between PSA, CPSE, and prostate volume

**Fig. 7:**
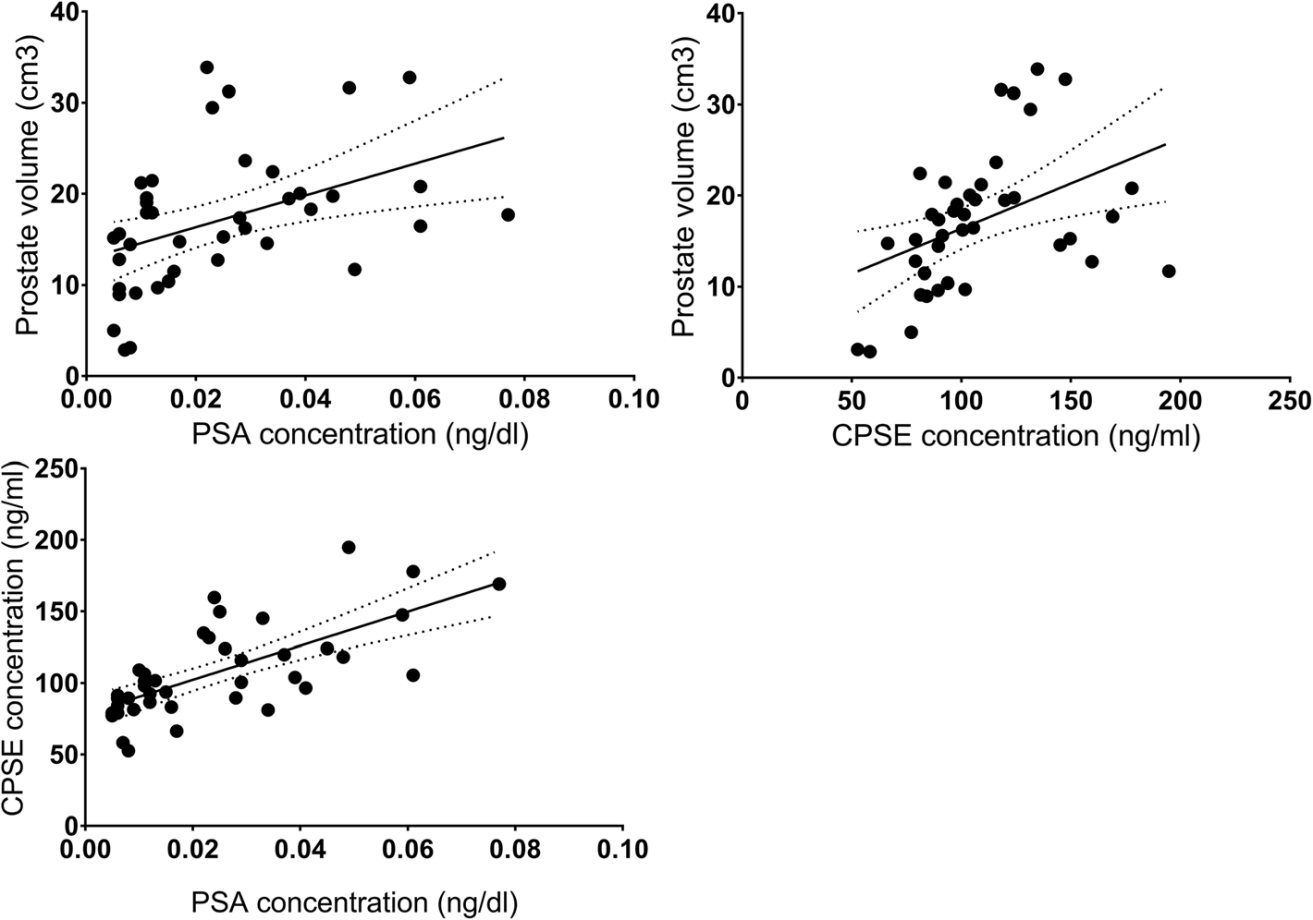
The correlation and regression were analyzed between the prostate volume and CPSE and PSA concentrations during the induction of PH and there was a significant positive correlation between the PSA concentration and prostate volume (Y = 174.7*X + 12.83; r^2^ = 0.18; *P* < 0.005) and between the CPSE level and prostate volume during the induction of PH in dogs (Y = 0.09936*X + 6.424; r^2^ = 0.18; *P* < 0.006). Also, there was a significant positive correlation between the PSA and CPSE levels during the induction of PH in dogs (Y = 1187X + 78.53; r^2^ = 0.68; *P* < 0.0001)

The correlation and regression were analyzed between the prostate volume and CPSE and PSA concentrations during the induction of PH, and there was a significant and positive correlation between the CPSE level and prostate volume during the induction of PH in dogs (Y = 0.09936*X + 6.424; r^2^ = 0.18; *P* < 0.006) and between PSA concentration and prostate volume (Y = 174.7*X + 12.83; r^2^ = 0.18; *P* < 0.005). Also, there was a significant positive correlation between the PSA and CPSE levels during the induction of PH in dogs (Y = 1187X + 78.53; r^2^ = 0.68; *P* < 0.0001) (Fig. 7).

### Hematological and serum biochemical parameters

The purposes of the measurement of hematological and serum biochemical factors were to monitor the general health of dogs during the induction of PH and the results confirmed that changes in the specific biomarkers were not under the influence of adverse effects caused by PH induction hormones. The results of the serum biochemical assay are presented in Table 2. There were no significant changes between groups or between the days of sampling in each group with regard to some of the factors including red cell distribution width (RDW), platelet, mean cell hemoglobin (MCH), mean corpuscular hemoglobin concentration (MCHC), monocytes, lymphocytes, bands, neutrophils, total protein, urea, sodium, cholesterol, and alkaline phosphatase (ALP) levels. The significant changes that were observed between the days of sampling in the control or PH-induced groups were in the normal range of each biochemical or hematological factor.

**Table 2:** Changes in the serum biochemicals during the induction of PH (63 days) in the control (*n* = 10) and PH-induced (*n* = 10) groups of intact dogs

Factor	Groups	Days			
		0	21	42	63
Glucose (mg/dl)	Control	62.7 ± 4.32^a^	87.3 ± 2.35^b^	87.7 ± 4.84*^b^	93.55 ± 3.85^b^
	PH Induction	65.55 ± 3.31^a^	86.65 ± 2.69^b^	67.9 ± 3.54*^a^	89.85 ± 1.6^b^
Urea (mg/dl)	Control	47.85 ± 11.32	30.05 ± 2.93	42.6 ± 2.76	40.2 ± 2.98
	PH Induction	43.95 ± 5.52	33.9 ± 2.68	43.89 ± 2.26	41.1 ± 3.08
Creatinine (mg/dl)	Control	1.022 ± 0.08^a^	0.9591 ± 0.04^b^	0.79 ± 0.06^b^	0.816 ± 0.05^b^
	PH Induction	1.074 ± 0.04^a^	0.997 ± 0.03	0.914 ± 0.05^b^	0.846 ± 0.06
TriGlycerides (mg/dl)	Control	45.7 ± 4.3^ac^	39.25 ± 2.88^a^	57.1 ± 5.34^c^	58.6 ± 4.42^c^
	PH Induction	37.4 ± 2.07^a^	41.75 ± 2.06	51.45 ± 3.01^b^	50.85 ± 4.07^b^
Cholesterol (mg/dl)	Control	187.9 ± 11.49	162.8 ± 15.18	168.6 ± 10.78	196.1 ± 11.92
	PH Induction	185.1 ± 13.75	173.4 ± 10.87	146.2 ± 10.2	169.5 ± 12.91
AST (U/L)	Control	29.63 ± 2.74^a^	16.56 ± 0.59^b^	20.26 ± 1.12^ab^	24.71 ± 2.86^ab^
	PH Induction	33.2 ± 1.97^a^	17.5 ± 0.98^b^	21.26 ± 2.83^b^	19.26 ± 1.61^b^
ALT (U/L)	Control	32.82 ± 4.6^a^	26.23 ± 1.54^a^	40.35 ± 4.5	50.65 ± 7.34^b^
	PH Induction	26.51 ± 2.71	24.98 ± 3.6	41.59 ± 6.05	40.7 ± 4.22
ALP (U/L)	Control	122.75 ± 20.02	115.5 ± 24.86	110.3 ± 21.02	120.95 ± 19.59
	PH Induction	106.2 ± 10.01	134.68 ± 16.83	131 ± 17.97	116.45 ± 15.78
Ca (mg/dl)	Control	11.06 ± 0.22^a^	9.83 ± 0.24^b^	9.97 ± 0.24^b^	9.89 ± 0.21^b^
	PH Induction	10.38 ± 0.21^a^	9.6 ± 0.14	9.36 ± 0.13 ^b^	9.77 ± 0.2
P (mg/dl)	Control	4.93 ± 0.2^a^	4.64 ± 0.24	3.96 ± 0.16^b^	4.65 ± 0.34
	PH Induction	5.21 ± 0.24	4.43 ± 0.16	4.46 ± 0.09	4.69 ± 0.21
Total protein (g/dl)	Control	5.13 ± 0.19	4.84 ± 0.2	4.5 ± 0.09	5.09 ± 0.26
	PH Induction	5.21 ± 0.24	4.73 ± 0.12	4.58 ± 0.11	4.72 ± 0.17
Albumin (g/dl)	Control	3.28 ± 0.11	2.84 ± 0.09	2.94 ± 0.12	2.86 ± 0.1
	PH Induction	3.41 ± 0.15^a^	2.77 ± 0.08^b^	2.72 ± 0.07^b^	2.76 ± 0.1^b^
Na (mEq/L)	Control	126.8 ± 1.91	127.8 ± 1.9	128.1 ± 1.96	136.5 ± 3.55
	PH Induction	132.5 ± 4.07	124.1 ± 1.62	124.9 ± 1.23	135.2 ± 5.4
K (mg/dl)	Control	3.81 ± 0.16	3.9 ± 0.16	3.33 ± 0.1	3.37 ± 0.12
	PH Induction	4.12 ± 0.22^a^	3.57 ± 0.08	3.26 ± 0.13^b^	3.4 ± 0.1^b^

^a,b,c^: For each factor, different superscript letters indicate significant differences in each row. * For each factor, different superscript letters indicate significant differences in each column

## Discussion

The canine model has been applied extensively for the investigation of prostate diseases, such as PH, due to its practical feasibility, efficiency, and safety [25, 26]. The key hormone in stimulating PH is dihydrotestosterone (DHT) in dogs, but its pathogenesis is not completely understood. In old dogs and dogs affected by PH, the prostatic volume has been larger. E2/T ratio and blood plasma concentrations of CPSE have been higher in the old dogs compared with those of the young dogs and dogs with normal prostate [27]. In the present study, a combination of estrogen and testosterone was used to induce PH, and the combination increased the prostatic volume based on the ultrasonographic examination.

Although dogs with PH tend to show higher serum CPSE concentrations compared with the normal dogs, the diagnostic efficacy of CPSE is restricted because its levels in dogs is affected by the prostatic carcinoma and prostatitis is not significantly different in dogs with PH. In addition, the concurrent PH with carcinoma or infection may influence the serum concentrations [28]. In another study, serum CPSE concentrations increased in dogs with PH. Thus, the evaluation can be performed to distinguish dogs affected by PH from healthy dogs [16]. Gobello et al. [20] claimed that CPSE appeared to be a promising discernment tool in non-tumoral canine prostatic diseases, but further investigation is essential to describe the precise function of CPSE. A study [29] revealed the positive relationships between the CPSE level and age as well as the CPSE level and the prostatic volume. In that study, no differences were observed in the prostatic volume of the groups although the volume of the prostate in large-sized dogs tended to be greater than that of the small-sized dogs. Moreover, other prostatic examinations of PH, such as digital palpation, prostate imaging, and the measurement of CPSE levels, may serve as useful methods for the differential diagnosis. A useful and accurate method for the diagnosis of PH in middle-aged dogs is the measurement of CPSE and it should be considered as an alternative or preferable tool to the conventional methods [30].

CPSE is a practical biomarker and can be used as a screening test in preventive programs for prostate diseases in dogs. In dogs without clinical signs, the CPSE serum levels greater than 50 ng/ml are correlated with ultrasonographic changes and increased prostatic volume (1.5 times greater than the normal volume) [31].

In another study, Along et al. 2018 [31] evaluated the role of CPSE in the early diagnosis of prostatic disorders and claimed that CPSE was an excellent tool for the diagnosis of prostatic diseases in a “prostate health screening program” before the patient was selected properly for more precise and costly methods of diagnoses. Our results showed that CPSE concentration increased significantly in the induction group compared to that of the control group. There was an almost steady trend in the CPSE level in the controls. Thus, based on our results and those of the previous studies, it could be stated that CPSE is an appropriate marker for the diagnosis of PH.

PSA concentration was measured and monitored during this study and a significant increase in the PSA levels was observed in comparison to those of the control. A positive and significant relationship was also observed between the PSA level and prostate volume and CPSE concentration. Amorium et al. 2004 measured PSA with a human commercial kit in the serum and urine samples of normal dogs, and the mean serum concentration of PSA was reported to be 0.005 ng/dl. The level of PSA in the serum of dogs was lower than humans (4–10 ng/ml) [32]. Bell et al. 1995 could not detect PSA in the canine serum or seminal plasma. PSA is irregularly present in the prostatic fluid and serum of both healthy and unhealthy dogs, affected by various prostatic disorders. Failure to detect PSA in the canine serum or seminal plasma suggests that either the concentration of PSA in these fluids is below the sensitivity of the assay used in the present study or no PSA is produced by the canine prostate gland [16].

There is acid phosphatase in dogs’ prostatic cells that is affected by hyperplastic and neoplastic changes, and also in normal dogs’ seminal plasma [16, 33]. The assaying of PAP and PSA in dogs is a new diagnostic test in veterinary medicine and their concentrations should be associated with the morphological changes in the prostate tissue [32]. In another survey, it was concluded that PAP levels did not differ according to the age and did not correlate with age or the prostatic diameter [34]. In our study, the PSA concentration dramatically increased in the induction group as it had been proven in other studies that PSA could be used for PH diagnosis. However, as regards PAP, it seems that more studies are needed to determine the importance of PAP in the PH diagnosis because no consistency was observed between the control and induction groups either in our study or other studies.

In the present study, hematologic factors, including RBC, Hgb, HCT, and MPV, fluctuated in the control and induction groups, following a decreasing trend. Eosinophil level and MCV in the control group were almost constant while their levels decreased in the induction group. WBC level was decreased in the control group while it increased in the induction group. Zinc is a significant trace element that adjusts the physiological growth of the prostate epithelium [35] Furthermore, the prostatic secretions contained calcium ions (Ca) and cholesterol that could infiltrate into the sperm through membrane fusion [12]. In dogs with PH, the motility and viability of sperm and secondary morphological abnormalities may be affected because of the changes in the biochemical factors in prostate secretions [35]. In our study, the biochemistry profiles and hematologic factors were measured for monitoring the function of liver and kidney, and there were no significant adverse changes. The glucose level in the control group showed an increasing trend while it was triglycerides concentration which increased in the induction group. The creatinine level in both groups decreased and other biochemical factors did not change significantly.

The biochemical factors of the prostatic secretions did not change in dogs affected by PH, but a significant difference was observed for the pH values [36]. There was no significant difference in the biochemical and hematological parameters, including urea, creatinine, total protein, albumin, and globulins, except in the elevation of serum acid phosphatase, C-reactive protein (CRP), and erythrocytes sedimentation rate (ESR) in some of the dogs in the PH group compared to the normal dogs. Moreover, the increase in serum acid phosphatase was more important than the increase in other factors [37]. The monitoring of hematological and biochemical factors was performed to help us determine whether there were any significant side effects of PH induction protocol on the general health of dogs.

## Conclusions

A combination of estrogen and testosterone was used to induce prostatic hyperplasia in dogs. Changes and of the increases in the serum CPSE and PSA concentrations and prostate volume were significant in the PH-induced dogs. A positive and significant correlation was observed between CPSE and PSA levels and between prostate volume and CPSE or PSA concentration in the PH-induced dogs. Measuring CPSE and PSA and ultrasonographic examination may be practical tools for the PH diagnosis in dogs. One of the limitations of the present study was the small number of animals per group that may have affected the explanation and interpretation of the results.

## Methods

### Animal ethics

Our study was submitted to and approved by Iranian animal ethics framework under the supervision of the Iranian Society for the Prevention of Cruelty to Animals and Shiraz University Research Council (IACUC no: 4687/63). The recommendations of European Council Directive (2010/63/EU) of September 22, 2010, regarding the standards in the protection of animals used for experimental purposes, were also followed.

### Animals

The dogs were kept in an NGO shelter in compliance with the standards of Shiraz University School of Veterinary Medicine for research purposes and all dogs were selected with a known history. The informed consent, written, was obtained from the shelter. All the dogs were castrated at the end of the study and kept in a shelter. Twenty adult male mixed-breed dogs, aged 2–3 years old and weighing 15–20 kg, were selected for this study. They were treated with antiparasitic drugs (one tablet of praziquantel for 10 kg/BW and one tablet of mebendazole for 5 kg/BW) for 2 weeks. All dogs were fed 300 g of commercial dog food (NUTRI® Dry Dog Food; Behintash Co. Iran) daily [38, 39]. The volume of each prostate was determined by ultrasonography examination. The volume was calculated based on length (L, cm), width (W, cm), and diameter (dorso-venteral distance), using the following equation: [(width × length × diameter) ÷2.6 + 1.8] [40]. The normal size of the prostate was estimated in conformity with the dogs’ weight (prostate volume (cm^3^) = (0.33 × body weight (kg) + 3.28)) [41]. The volume of the prostate which is calculated should not exceed the normal size based on the body weight.

### Protocol

After deworming, animals were randomly assigned to two equal PH induction and control groups (*n* = 10). In the PH induction group, dogs received testosterone enanthate (Caspian Tamin, Iran; 75 mg/dog) and estradiol benzoate (Aburaihan, Iran; 0.75 mg/dog) via intramuscular injection on days 0, 21, 42, and 63. The testosterone doses were doubled on days 21 and 42 [38, 39, 42]. Blood sampling was performed from the jugular vein into glass tubes. They were centrifuged for 10 min at 750×g and the serum was stored at − 20 °C until measurement. Ultrasonographic examination, prostatic biomarkers, and biochemistry profiles were submitted on days 0, 21, 42, and 63. Hematologic factors were submitted on days 0 and 63.

### Laboratory biochemical and hormone assays

Briefly, the commercial enzyme-linked immunosorbent assay (ELISA) kits were used to measure canine prostatic specific esterase (CPSE) (Odelis CPSE, Virbac BVT, France); serum prostatic specific antigen (PSA) (M&F Biotech Co., Ltd., Hangzhou, China); serum testosterone (CUSABIO, Wuhan Hi-tech Medical Devices Park, Wuhan, Hubei Province, China) and serum dihydrotestosterone (DHT) (Zhuhaishi Shuangbojie Technology Co., Ltd). Canine prostatic acid phosphatase (PAP) was measured using a commercial kit (Biotrol Pac monoreactif, A03034, Biotrol Diagnostic, France) and colorimetric automation equipment (Alpha Classic AT++, Sanjesh, Iran) [38].

The different hematological parameters were measured using a veterinary hematology analyzer (Nihon Kohden, MEK-6450 Celltac Alpha, Tokyo, Japan). The differential leukocyte counts were determined with preparing and staining a blood smear on a glass slide. Serum biochemical parameters were measured using standard methods and commercial kits (Pars Azmoon Co., Tehran, Iran), and a biochemical auto analyzer (Alpha Classic AT++, Sanjesh, Iran). A flame photometer apparatus (Fater Electron Company, Tehran, Iran) was used to measure serum concentration of sodium and potassium [38].

### Statistical analysis

Data were statistically analyzed with SPSS version 16 (IBM, CA). Two-way ANOVA, Bonferroni’s multiple comparison tests, and group and time factors were also utilized for detailed analysis of data. Regression analysis was performed to evaluate the relationships between prostate volume and CPSE and PSA concentrations and between CPSE and PSA levels. The changes (%) in serum biomarkers were calculated as (B-A)/A × 100 (A: data on day 0, B: data on day 63 of study). In all the analyses, a *P* < 0.05 was considered as significant.

## Data Availability

Datasets generated and/or analyzed during the current study are available in the figshare (https://figshare.com/s/9f42e3f4602122b304fc).

## Abbreviations

ALP: Alkaline phosphatase
ALT: Alanine aminotransferase
ANOVA: Analysis of variance
AST: Aspartate amino transferase
CPSE: Canine prostate-specific esterase
DHT: Dihydrotestosterone
ELISA: Enzyme-linked immunosorbent assay
MCH: Mean cell hemoglobin
MCHC: Mean corpuscular hemoglobin concentration
PAP: Prostatic acid phosphatase
PH: Prostatic hyperplasia
PSA: Prostate-specific antigen
RDW: Red cell distribution width
T: Testosterone
